# Mediterranean diet and quality of life: Baseline cross-sectional analysis of the PREDIMED-PLUS trial

**DOI:** 10.1371/journal.pone.0198974

**Published:** 2018-06-18

**Authors:** Iñigo Galilea-Zabalza, Pilar Buil-Cosiales, Jordi Salas-Salvadó, Estefanía Toledo, Carolina Ortega-Azorín, Javier Díez-Espino, Zenaida Vázquez-Ruiz, María Dolores Zomeño, Jesús Vioque, José Alfredo Martínez, Dora Romaguera, Napoleón Perez-Farinos, José López-Miranda, Ramón Estruch, Aurora Bueno-Cavanillas, Fernando Arós, Josep Antoni Tur, Francisco Tinahones, Lluis Serra-Majem, Alba Marcos-Delgado, Manuel Ortega-Calvo, Clotilde Vázquez, Xavier Pintó, Josep Vidal, Lidia Daimiel, Miguel Delgado-Rodríguez, Pilar Matía, Dolores Corella, Andrés Diaz-López, Nancy Babio, Miguel Angel Muñoz, Montse Fitó, Sandra González-Palacios, Itziar Abete, Antonio García-Rios, Emilio Ros, Miguel Ángel Martínez-González, for the PREDIMED-PLUS Study Investigators

**Affiliations:** 1 Atención Primaria. Osasunbidea-Servicio Navarro de Salud. Pamplona, Spain; 2 CIBER Fisiopatología de la Obesidad y Nutrición (CIBERobn), Instituto de Salud Carlos III (ISCIII), Madrid, Spain; 3 Department of Preventive Medicine and Public Health, University of Navarra-IdiSNA, Pamplona, Spain; 4 Human Nutrition Unit, IISPV, Universitat Rovira i Virgili, Reus, Spain; 5 Department of Preventive Medicine, University of Valencia, Valencia, Spain; 6 Cardiovascular Risk and Nutrition, IMIM-Hospital del Mar Medical Research Institute, Barcelona, Spain; 7 Blanquerna School of Life Sciences, Universitat Ramon Llull, Barcelona, Spain; 8 CIBER Epidemiología y Salud Pública (CIBEResp), Instituto de Salud Carlos III (ISCIII), Madrid, Spain; 9 Nuritional Epidemiology Unit, Miguel Hernandez University, ISABIAL-FISABIO, Alicante, Spain; 10 Department of Nutrition and Food Sciences, Physiology and Toxicology, University of Navarra, Pamplona, Spain; 11 Health Research Institute of the Balearic Islands (IdISBa), University Hospital Son Espases, Palma, Spain; 12 School of Nursing, University of Málaga, Málaga, Spain; 13 Department of Internal Medicine, Reina Sofia University Hospital, University of Córdoba-IMIBIC, Córdoba, Spain; 14 Department of Internal Medicine, IDIBAPS, Hospital Clinic, University of Barcelona, Barcelona, Spain; 15 Department of Preventive Medicine, University of Granada, Granada, Spain; 16 Department of Cardiology OSI ARABA. University Hospital Araba, Vitoria, Spain; 17 University of the Basque Country UPV/EHU, Vitoria-Gasteiz. Spain; 18 Department of Endocrinology, University Hospital, University of Málaga, Málaga, Spain; 19 Institute for Biomedical Research, University of Las Palmas de Gran Canaria, Las Palmas, Spain; 20 Instituto de Biomedicina (IBIOMED); Universidad de León, León, Spain; 21 Department of Family Medicine, Distrito Sanitario Atencion Primaria, Centro de Salud Las Palmeritas, Sevilla, Spain; 22 Department of Endocrinology, Fundación Jiménez-Díaz, Madrid, Spain; 23 Lipids and Vascular Risk Unit, Internal Medicine, Hospital Universitario de Bellvitge, Hospitalet de Llobregat, Barcelona, Spain; 24 CIBER Diabetes y enfermedades metabólicas (CIBERdem), Instituto de Salud Carlos III (ISCIII), Madrid, Spain; 25 Department of Endocrinology, IDIBAPS, Hospital Clinic, University of Barcelona, Barcelona, Spain; 26 Nutritional Genomics and Epigenomics Group, IMDEA Food, CEI UAM + CSIC, Madrid, Spain; 27 Division of Preventive Medicine, University of Jaén, Jaén, Spain; 28 Instituto de Investigación Sanitaria del Hospital Clínico San Carlos, Madrid, Spain; 29 Lipids and Cardiovascular Epidemiology Research Unit, Institut Municipal d’Investigació Mèdica (IMIM), Barcelona, Spain; 30 Lipid Clinic, Department of Endocrinology and Nutrition, Institut d’Investigacions Biomèdiques August Pi Sunyer (IDIBAPS), Hospital Clínic, Barcelona, Spain; 31 Department of Nutrition, Harvard T. H. Chan School of Public Health, Boston, United State of America; University of Zurich, SWITZERLAND

## Abstract

We assessed if a 17-item score capturing adherence to a traditional Mediterranean diet (MedDiet) was associated with better health-related quality of life among older Spanish men and women with overweight or obesity harboring the metabolic syndrome. We analyzed baseline data from 6430 men and women (age 55–70 years) participating in the PREDIMED-Plus study. PREDIMED-Plus is a multi-centre randomized trial testing an energy-restricted MedDiet combined with promotion of physical activity and behavioral therapy for primary cardiovascular prevention compared to a MedDiet alone. Participants answered a 36-item questionnaire about health-related quality of life (HRQoL) and a 17-item questionnaire that assessed adherence to an MedDiet. We used ANCOVA and multivariable-adjusted linear regression models to compare baseline adjusted means of the quality of life scales according to categories of adherence to the MedDiet. Higher adherence to the MedDiet was independently associated with significantly better scores in the eight dimensions of HRQoL. Adjusted differences of > = 3 points between the highest and the lowest dietary adherence groups to the MedDiet were observed for vitality, emotional role, and mental health and of > = 2 points for the other dimensions. In conclusion, this study shows a positive association between adherence to a MedDiet and several dimensions of quality of life.

## Introduction

Even though numerous studies have linked a high-quality dietary pattern with lower incidence of chronic diseases [1], particularly cardiovascular disease [2,3], the effect of high-quality dietary patterns on health-related quality of life (HRQL) is still not well known [4–7]. Self-perceived HRLQ is a relevant variable because it could be a predictor of chronic disease and mortality in the long term [8–9] and it is likely to be influenced by an overall high-quality dietary pattern.

During the last 2 decades, the use of patient-reported outcome measures represents a strong shift in medicine, because previously the main reliance was on clinical measurements and biomarkers instead of self-reported information. In this context, self-reported quality of life is considered as the extent to which the life of a person is felt as comfortable or satisfying. Quality of life has more to do with happiness, convenience, well-being and easiness in life than with wealth, power or hierarchical/professional roles. According to the US government goals “Healthy People 2020”, health-related quality of life (HRQL) is a “multi-dimensional concept that includes domains related to physical, mental, emotional, and social functioning. It goes beyond direct measures of population health, life expectancy, and causes of death, and focuses on the impact health status has on quality of life” (www.healthypeople.gov). The most frequently used tool to appraise HRQL is the short-form 36 questionnaire.

HRQL can be partly determined by dietary patterns. Studying dietary patterns instead of individual foods or nutrients is the state-of-the-art in current nutritional epidemiology, because complete dietary patterns provide a more realistically picture of food consumption habits while capturing the synergistic or antagonistic effects that foods and nutrients may have when they are consumed together [10]. In the context of overall dietary patterns, the traditional Mediterranean diet (MedDiet) has been found to be associated with better HRQL in the Moli-sani Project, a population-based cohort study in Italy [11]. Smaller cross-sectional studies conducted in Spain [12–13] have also shown positive associations between adherence to the MedDiet and HRQL. In the SUN Project [14], a Spanish multipurpose prospective cohort study including more than 11,000 middle-aged university graduates, a significant direct association between higher baseline adherence to the MedDiet and better physical and mental health dimensions of HRQL was reported after a 4-year follow-up.

In general, studies have found better HRQL associated with higher adherence to the Mediterranean diet, but the dimensions of health categories differ among studies. In some studies, the beneficial association was observed for the physical dimensions [7,14], whereas in others improvements were observed only for mental dimensions [11,13,15]. The ascertainment of the reasons for these differences and the mechanisms involved is still a matter of investigation.

HRQL is becoming more important with the steady increase in life expectancy and could inform general practitioners and policy makers for decision-making. Collecting sound evidence on the link between high adherence to an MedDiet and HRQL among older individuals at high risk of cardiovascular disease is especially appealing from a public health perspective. In this context, the primary aim of our study was to assess whether baseline adherence to a MedDiet was cross-sectionally associated with better baseline HRQL in the participants of the PREDIMED-Plus randomized trial.

## Methods

### Study design and participants

We performed a cross-sectional analysis of the participants in the PREDIMED-Plus study, a multi-centre randomized trial. PREDIMED-Plus aims to evaluate the effect of an energy-restricted MedDiet associated with physical exercise and behavioral therapy compared to a traditional Mediterranean diet alone for the primary prevention of cardiovascular disease in Spain (http://medpreventiva.es/QufSWn). The trial was approved by de Institutional Review Board of all the recruitment centers where the study was conducted (CEI Provincial de Málaga, CEI de los Hospitales Universitarios Virgen Macarena y Virgen del Rocio, CEI de la Universidad de Navarra, CEI de las Illes Balears, CEIC del Hospital Clinic de Barcelona, CEIC del Parc de Salut Mar, CEIC del Hospital Universitari Sant Joan de Reus, CEI del Hospital Universitario San Cecilio, CEIC de la Fundacion Jimenez Dıaz, CEIC Euskadi, CEI en Humanos de la Universidad de Valencia, CEIC del Hospital Universitario de Gran Canaria Doctor Negrın, CEIC del Hospital Universitario de Bellvitge, CEI de Cordoba, CEI de Instituto Madrileño De Estudios Avanzados, CEIC del Hospital Clınico San Carlos, CEI Provincial de Malaga, CEI de las Illes Balears, CCEI de la Investigacion Biomedica de Andalucıa and CEIC de Leon.

Participants signed a written informed consent form. The trial was registered in 2014 at the International Standard Randomized Controlled Trial (ISRCTN89898870) and it was funded by the European Research Council (Advanced Research Grant (http://medpreventiva.es/N37jk1).

Participants in the PREDIMED-Plus trial are men aged between 55–75 years and women aged between 60–75 years, with a body mass index (BMI) between 27 and 40 kg/m^2^ and disclosing the metabolic syndrome [16], but no cardiovascular disease at enrollment.

**Fig 1:**
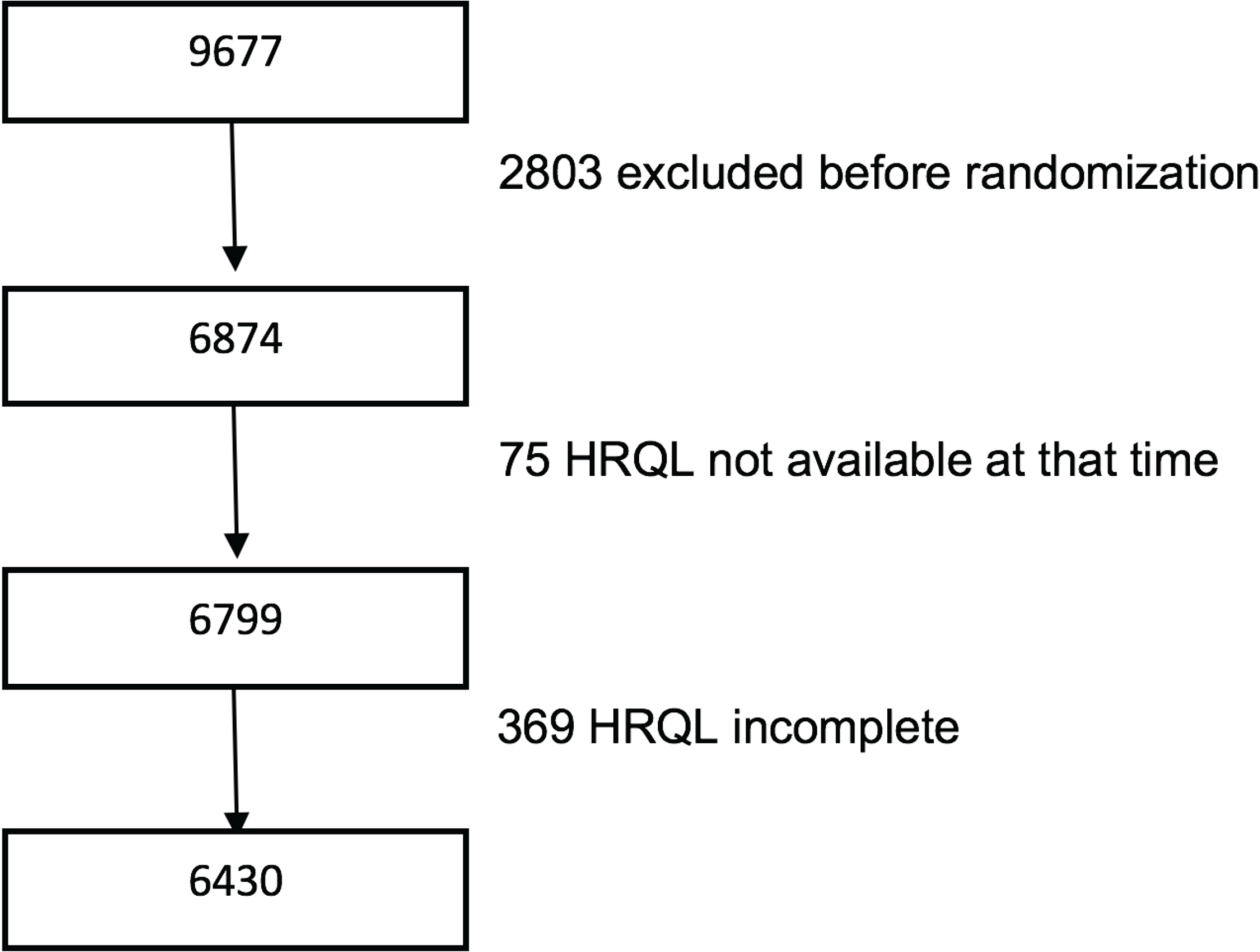
Flow-chart of participants in the PREDIMED Plus trial. HRQL: Health related quality of life.

The recruitment took place between September 2013 and December 2016. During that period, 6874 participants were recruited and randomized. For the present analysis, we included all participants who had answered the adapted HRQL questionnaire validated for the Spanish population [14,15], and the 17-item questionnaire of adherence to a MedDiet (Table 1). In total, 6768 participants answered to the SF-36, but only 5416 among them completed all the items of the questionnaire. As some participants left blank only some items of the questionnaire, we imputed for these 1352 participants the missing items using their average score for the other items belonging to that same dimension. Thus, we included information on 6430 participants (Fig 1).

**Table 1:** Mediterranean diet used in the intervention arm of the PREDIMED-PLUS trial: 17-point questionnaire to assess adherence.

Questions	Criteria for 1 point
Do you use only extra-virgin olive oil for cooking, salad dressings, and spreads?	Yes
How many fruit units (including natural fruit juices) do you consume per day?	≥3
How many servings of vegetables/garden produce do you consume per day? [1 serving: 200 g (consider side dishes as half a serving)]	≥2 (≥1 portion raw or in a salad)
How many servings of white bread do you consume per day? (1 serving: 75 g)	≤1
How many times per week do you consume whole grain cereals and pasta?	≥5
How many servings of red meat, hamburgers, or meat products (ham, sausage, etc.) do you consume per week? (1 serving: 100–150 g)	≤1
How many servings of butter, margarine, or cream do you consume per week? (1 serving: 12 g)	<1
How many sugary beverages or sugar-sweetened fruit juices do you drink per week?	<1
How many servings of legumes do you consume per week? (1 serving: 150 g)	≥3
How many servings of fish or shellfish do you consume per week? (1 serving: 100–150 g of fish or 4–5 units or 200 g of shellfish)	≥3
How many times per week do you consume commercial sweets or pastries (not homemade), such as cakes, cookies, sponge cake, or custard?	<3
How many servings of nuts (including peanuts) do you consume per week? (1 serving: 30 g)	≥3
Do you preferentially consume chicken, turkey or rabbit instead of beef, pork hamburgers or sausages?	Yes
How many times per week do you consume vegetables, pasta, rice or other dishes seasoned with *sofrito* (sauce made with tomato and onion, leek or garlic and simmered in olive oil)?	≥2
Do you preferentially add non-caloric artificial sweeteners to beverages (such as coffee or tea) instead of sugar?	Yes
How many times per week do you consume non-whole grain pasta or white rice?	<3
How many glasses of wine do you drink per day? (1 glass: 100 ml)	2–3 for men 1–2 for women

### Diet

**Fig 2:**
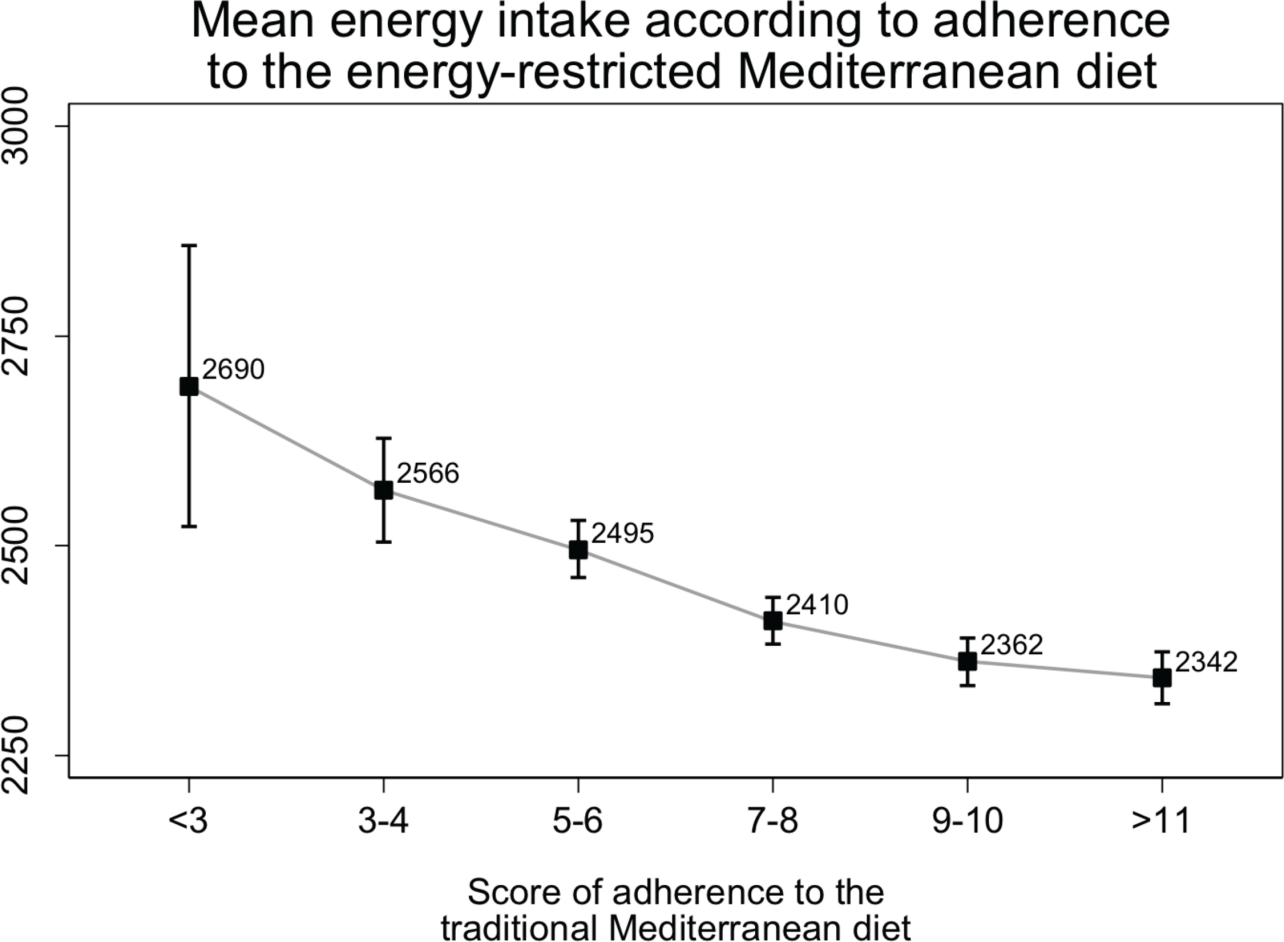
Mean energy intake according to adherence to the Mediterranean diet.

The traditional Mediterranean diet is characterized by the use of olive oil as main culinary fat and, consequently, a high intake of fat from vegetable sources and of fruits, vegetables, legumes, nuts and fish, and a low intake of red meat and sweets. In the 17-item screener shown in Table 1 that we developed to assess adherence to a MedDiet, an adequate consumption of typical traditional Mediterranean foods adds one point and low consumption of foods not characteristic of the MedDiet diet also adds one point. In this 17-item questionnaire we took into account the need for weight loss among these overweight/obese participants and included several items (#4, #5, #6–8, #11, #15–16) specifically tailored to improve the ability of the MedDiet to attain long-term sustainable weight loss. An, in fact, an inverse relationship was observed between better adherence to the 17-item MedDiet screener and total energy intake (Fig 2). All participants completed the 17-item screener in the baseline visit.

### Health-related quality of life

We used an adapted version of previously published questionnaires of HRQL that were validated for the Spanish population [7,14]. This questionnaire has been extensively used in Spain as an accurate way to measure self-perceived HRQL (**https://goo.gl/Uyn5u6, https://goo.gl/Gs31ua**). It allows studying different dimensions that can be grouped in an aggregated physical dimension and an aggregated mental dimension. Specifically, the 36 items measure eight multi-item dimensions: physical functioning, role limitations due to physical health problems (role-physical), bodily pain, general health perceptions, vitality, social functioning, role limitations due to emotional problems (role emotional) and mental health. The first four domains deal with physical aspects, and the next four reflect psychological features. For each parameter, scores are coded, summed and transformed to a scale from 0 (the worst possible condition) to 100 (the best possible condition). This questionnaire is a useful tool to compare health status that varies according to different healthy lifestyles and diseases [17]. The HQRL was completed during the baseline visit.

### Other covariates

Only participants with metabolic syndrome were included in the PREDIMED-Plus trial. After an overnight fasting, blood samples were collected at the initial screening visits. Aliquots of serum and EDTA plasma were immediately processed, coded and stored at -80°C in a central laboratory until analysis. Serum glucose, triglyceride, total and high-density lipoprotein (HDL) cholesterol levels were measured by routine laboratory tests using standard enzymatic methods. Blood pressure was measured using a validated semiautomatic oscillometer (Omron HEM-705CP, Netherlands) after 5 minutes of rest in-between measurements. Height, waist circumference and weight were measured at baseline by trained staff. Body-mass index was calculated as weight (kg) over height squared (m^2^). All anthropometric variables were determined in duplicate, except for blood pressure (in triplicate). The cut-off points used to define metabolic syndrome (> = 3 criteria over 5 criteria) were fasting glucose> = 100 mg/dl, triglycerides> = 150 mg/dl, HDL<40 mg/dl in men or <50 in women and blood pressure > = 130/85 mm Hg. In the Caucasian population, the cut-off point defining the abdominal obesity criterion for metabolic syndrome was > = 80 cm in women and > = 94 cm in men. In the South American population, the value is the same for women but for men It was > = 90 cm.

Information about age, sex, physical activity (measured in METs-minutes/week), civil status and educational level was collected with a general questionnaire prior to randomization.

For measuring physical activity, we used the short form of the Minnesota Leisure Time Physical Activity Questionnaire validated for the Spanish population [18,19].

We defined cases of high blood pressure, diabetes, history of depression, chronic lung disease and cancer as the respective diagnosis received by a physician.

### Statistical analysis

According to the 17-item questionnaire, we categorized baseline adherence to the MedDiet into approximate quartiles, defining the four groups as “Low” (0–6 points), “Low to Moderate” (7–8 points), “Moderate to High” (9–10 points), and “High (11–17)”. Baseline characteristics were described as means (standard deviations) for quantitative traits and as proportions for qualitative traits according to the baseline adherence to categories of the MedDiet. We compared the means in each HRQL dimension (and the 2 aggregated dimensions) across the four levels of adherence to the MedDiet with ANCOVA models and calculated the between-group differences with linear regression models. We fitted three different models for each dimension provided by the HRQL questionnaire. The first model was adjusted for sex, age and recruitment centre. A second model included additional adjustments for body-mass index, physical activity (METs-min/week), smoking status (never smoker / former smoker/ current smoker), marital status (single / married / divorced / widowed), highest level of education attained (primary school or less / Secondary studies / College graduate). A third model was also adjusted for comorbidities that have been associated with poorer quality of life such as high blood pressure (yes/no), diagnosis of type-2 diabetes (yes/no) history of depression (yes/no), chronic lung disease (yes/no) and cancer (yes/no).

We also assessed the linear trends for the association between baseline adherence to the MedDiet and the different dimensions of the HRQL, for this aim, we tested the 17-item score as a continuous quantitative variable included in the multivariable-adjusted models. We considered a two-tailed value of 0.05 as threshold for statistical significance. We conducted all the analyses with Stata (Stata/SE 15.1 StataCorp College Station, Texas).

## Results

Baseline characteristics of the PREDIMED-Plus participants are shown in Table 2 according to categories of baseline adherence to the MedDiet. Mean age ranged from 64.2 years in the “Low” adherence group to 65.5 years in the “High” adherence group. In the “High” adherence group there were more women and participants with diabetes, and higher levels of physical activity (METs-min/week). There were also less former smokers and a lower prevalence of hypertension, and the average body-mass index was slightly lower.

**Table 2:** Baseline characteristics of the participants in the PREDIMED-Plus trial according to baseline categories (roughly quartiles) of adherence to the traditional Mediterranean diet.

	Adherence to the Mediterranean diet			
	Low	Low-medium	Medium-high	High
Score [MedDiet (0 to 17)]	0–6	7–8	9–10	11–17
**N**	1567	1730	1647	1486
**Energy consumption (Kcal/d)**	2553(670)	2404(636)	2347(575)	2312(583)
**Age (years)**	64 (5)	65 (5)	65 (5)	66 (5)
**Female sex (%)**	37.4	47.6	50.5	56.5
**Body-mass index (Kg/m**^**2**^**)**	32.9 (3.4)	32.8 (3.5)	32.8 (3.4)	32.4 (3.4)
**Physical activity (METs-min/week)**	2187(2189)	2360(2159)	2644(2431)	2972(2628)
**Diabetes at baseline (%)**	22.2	27.6	28.8	29.5
**Depression at baseline (%)**	18.6	21.1	20.5	22.1
**Hypertension at baseline (%)**	84.8	84.0	84.0	81.2
**History of cancer (%)**	7.3	7.4	6.6	7.5
**History of lung disease (%)**	4.3	4.1	4.7	4.5
**Smoking status (%)**				
**Current smoker**	15.7	13.6	10.9	9.6
**Former smoker**	44.9	43.5	42.9	44.4
**Never smoker**	39.4	42.9	46.1	46.1
**Marital status (%)**				
**Married**	77.5	77.2	77.4	74.4
**Single**	5.2	4.5	5.2	5.5
**Divorced**	7.4	8.1	7.7	8.8
**Widowed/widower**	9.8	10.2	9.8	11.4
**Maximum attained educational level (%)**				
**Primary school or less**	46.7	49.9	48.3	47.8
**Secondary school**	32.0	31.0	28.4	26.4
**College or higher**	21.3	19.1	22.3	25.8
**Anti-platelet therapy (%)**	13.6	14.7	16.7	17.4
**Blood-pressure lowering therapy (%)**	78.2	77.3	77.6	76.2
**Lipid lowering therapy (%)**	49.6	49.8	49.8	51.6
**Insulin therapy (%)**	4.2	4.5	5.1	4.9
**Metformin therapy (%)**	18.6	23.1	24.0	24.0
**Other oral antidiabetic therapy (%)**	15.4	20.2	20.1	19.5

MedDiet: Mediterranean diet

Data are presented as mean (standard deviation) or percentage.

Tables 3 and 4 show the mean scores in the different dimensions of HRQL according to categories of baseline adherence to the MedDiet. In the fully adjusted models, we observed that a higher adherence to the MedDiet was associated with better scores in all dimensions of HQRL. When the individual dimensions were aggregated, a higher adherence to the MedDiet was also associated with a better score in the aggregated mental and physical dimension.

**Table 3:** Adjusted means for each of the 8 dimensions of health-related quality of life by baseline categories of adherence to a traditional Mediterranean diet. The PREDIMED-Plus trial.

Physical Role					
	**Adherence to tde traditional Mediterranean diet**				
	**<7**	**7–9**	**9–10**	**11–17**	**P for trend**
**N**	**1567**	**1730**	**1647**	**1486**	
**Age & sex adjusted**	73.84 (72.08 to 75.60)	75.75 (74.13 to 77.37)	77.25 (75.59 to 78.91)	77.63 (75.84 to 79.42)	<0.001
Multivariable adjusted 1*	74.49 (72.73 to 76.26)	76.17 (74.56 to 77.78)	77.08 (75.43 to 78.73)	76.64 (74.84 to 78.43)	0.064
Multivariable adjusted 2**	74.39 (72.66 to 76.14)	76.15 (74.56 to 77.74)	77.07 (75.44 to 78.70)	76.78 (75.01 to 78.56)	0.043
**Bodily Pain**					
	**<7**	**7–9**	**9–10**	**11–17**	**P for trend**
**Age & sex adjusted**	60.82 (59.53 to 62.14)	61.42 (60.22 to 62.61)	62.56 (61.33 to 63.78)	64.16 (62.85 to 65.49)	<0.001
Multivariable adjusted 1*	61.26 (59.96 to 62.55)	61.75 (60.56 to 62.93)	62.47 (61.25 to 63.68)	63.44 (62.11 to 64.75)	0.011
Multivariable adjusted 2**	61.15 (59.87 to 62.43)	61.74 (60.57 to 62.90)	62.49 (61.29 to 63.68)	63.53 (62.23 to 64.83)	0.006
**General Health**					
	**<7**	**7–9**	**9–10**	**11–17**	**P for trend**
**Age & sex adjusted**	60.70 (59.76 to 61.64)	61.77 (60.91 to 62.63)	62.85 (61.97 to 63.73)	64.03 (63.08 to 64.98)	<0.001
Multivariable adjusted 1*	61.28 (60.36 to 62.22)	62.10 (61.25 to 62.94)	62.68 (61.81 to 63.55)	63.21 (62.27 to 64.16)	0.002
Multivariable adjusted 2**	60.93 (60.03 to 61.84)	62.10 (61.27 to 62.92)	62.82 (61.98 to 63.68)	63.42 (62.50 to 64.34)	<0.001
**Physical function**					
	**<7**	**7–9**	**9–10**	**11–17**	**P for trend**
**Age & sex adjusted**	74.54 (73.61 to 75.47)	75.15 (74.30 to 76.01)	76.53 (75.65 to 77.41)	78.53 (77.58 to 79.47)	<0.001
Multivariable adjusted 1*	75.20 (74.30 to 76.10)	75.59 (74.77 to 76.41)	76.39 (75.55 to 77.24)	77.47 (76.56 to 78.39)	<0.001
Multivariable adjusted 2**	75.16 (74.27 to 76.04)	75.59 (74.78 to 76.40)	76.39 (75.56 to 77.21)	77.53 (76.63 to 78.43)	<0.01
**Vitality**					
	**<7**	**7–9**	**9–10**	**11–17**	**P for trend**
**Age & sex adjusted**	61.05 (60.00 to 62.10)	62.88 (61.92 to 63.45)	64.17 (63.18 to 65.16)	66.09 (65.03 to 67.16)	<0.001
Multivariable adjusted 1*	61.80 (60.77 to 62.83)	63.27 (62.33 to 64.21)	63.94 (62.97 to 64.91)	65.10 (64.05 to 66.15)	<0.001
Multivariable adjusted 2**	61.59 (60.58 to 62.59)	63.28 (62.36 to 64.20)	64.00 (63.05 to 64.93)	65.26 (64.23 to 66.28)	<0.001
**Social function**					
	**<7**	**7–9**	**9–10**	**11–17**	**P for trend**
**Age & sex adjusted**	84.21 (83.17 to 85.26)	85.58 (84.62 to 86.54)	86.37 (85.39 to 87.36)	87.53 (85.39 to 87.36)	<0.001
Multivariable adjusted 1*	84.69 (83.65 to 85.73)	85.82 (84.86 to 86.77)	86.21 (85.23 to 87.19)	86.93 (85.83 to 88.99)	0.005
Multivariable adjusted 2**	84.54 (83.52 to 85.56)	85.82 (84.90 to 86.76)	86.21 (85.26 to 87.17)	87.08 (86.05 to 88.12)	0.001
**Emotional role**					
	**<7**	**7–9**	**9–10**	**11–17**	**P for trend**
**Age & sex adjusted**	84.09 (82.55 to 85.62)	86.64 (85.23 to 88.05)	88.04 (86.59 to 89.49)	88.26 (86.07 to 89.82)	<0.001
Multivariable adjusted 1*	84.53 (82.99 to 86.06)	86.85 (85.44 to 88.25)	87.87 (86.43 to 89.31)	87.75 (86.18 to 89.32)	<0.001
Multivariable adjusted 2**	84.36 (82.85 to 85.87)	86.88 (85.50 to 88.25)	87.84 (86.42 to 89.25)	87.92 (86.39 to 89.45)	<0.001
**Mental Health**					
	**<7**	**7–9**	**9–10**	**11–17**	**P for trend**
**Age & sex adjusted**	72.37 (71.42 to 73.32)	73.89 (73.03 to 74.77)	75.13 (74.24 to 76.03)	76.28 (75.32 to 77.24)	<0.001
Multivariable adjusted 1*	72.88 (71.94 to 73.82)	74.13 (73.27 to 74.99)	74.95 (74.07 to 75.83)	75.67 (74.71 to 76.53)	<0.001
Multivariable adjusted 2**	72.69 (71.78 to 73.61)	74.14 (73.31 to 74.97)	74.99 (74.13 to 75.84)	75.81 (74.89 to 76.74)	<0.001

*Additionally adjusted for body mass index (kg/m^2^), physical activity (METs-min/week), smoking status (never, former, current), civil status (single, married, divorced, widowed), educational level (primary school or less, secondary, college or higher)

**Additionally adjusted for high blood pressure, diabetes (dichotomous),depression, chronic obstructive lung disease, and cancer prevalence.

**Table 4:** Adjusted means for the 2 aggregated dimensions of health-related quality of life by baseline categories of adherence to a traditional Mediterranean diet. The PREDIMED-Plus trial.

**Aggregated Physical dimensions**					
	**<7**	**7–9**	**9–10**	**11–17**	**P for trend**
**Age & sex adjusted**	44.44 (44.00 to 44.88)	44.62 (44.22 to 45.02)	45.06 (44.65 to 45.46)	45.68 (45.23 to 46.12)	0,001
Multivariable adjusted 1*	44.69 (44.26 to 45.12)	44.78 (44.39 to 45.17)	45.06 (44.61 to 45.41)	45.28 (44.85 to 45.71)	0.045
Multivariable adjusted 2**	44.63 (44.20 to 45.05)	44.78 (44.39 to 45.16)	45.03 (44.63 to 45.43)	45.33 (44.90 to 45.76)	0. 019
**Aggregated Mental dimensions**					
	**<7**	**7–9**	**9–10**	**11–17**	**P for trend**
**Age & sex adjusted**	50.12 (49.60 to 50.64)	51.11 (50.64 to 51.59)	51.63 (51.14 to 52.12)	52.00 (51.47 to 52.53)	<0.001
Multivariable adjusted 1*	50.33 (49.81 to 50.85)	51.20 (50.72 to 51.67)	51.54 (51.05 to 52.03)	51.78 (51.25 to 52.31)	<0.001
Multivariable adjusted 2**	50.25 (49.74 to 50.75)	51.21 (50.75 to 51.67)	51.55 (51.07 to 52.02)	51.86 (51.34 to 52.37)	<0.001

*Additionally adjusted for body mass index (kg/m^2^), physical activity (METs-min/week), smoking status (never, former, current), civil status (single, married, divorced, widowed), educational level (primary school or less, secondary, college or higher)

**Additionally adjusted for high blood pressure, diabetes (dichotomous),depression, chronic obstructive lung disease, and cancer prevalence.

**Fig 3:**
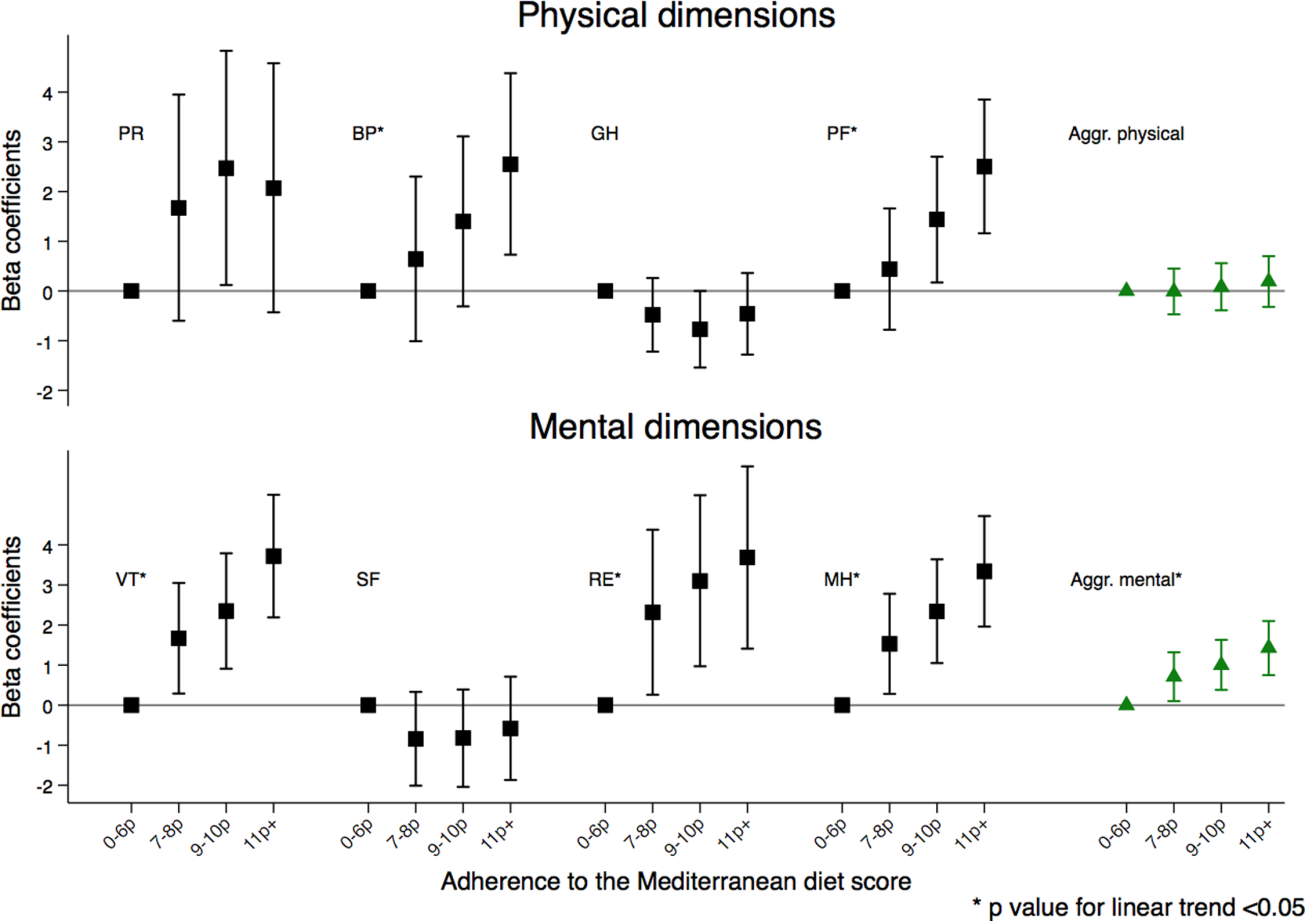
Mean differences in the health-related quality of life dimensions according to baseline adherence to the traditional Mediterranean diet in the PREDIMED-Plus trial. PR-Physical role BP-Bodily pain GH-General health PF-Physical functioning VT-Vitality SF- Social function RE-Emotional Role MH-Mental Health Aggr Physical Physical aggregated Aggr Mental- Mental Aggregated.

Fig 3 shows mean differences and confidence intervals in the different individual and aggregated dimensions for the categories of increasing adherence to the MedDiet diet compared to the lowest category. Differences of at least three points between the high and the low adherence groups were observed for vitality, emotional role and mental health and of at least two points for the other dimensions in the fully adjusted models.

## Discussion

We found a direct association between baseline adherence to an MedDiet and all dimensions of health-related quality of life. Beyond merely statistically significant differences, clinically meaningful associations were also observed, because we found differences greater than three points in three of them, belonging to mental health dimensions [20–23]. Our results are consistent with previous findings. [7,11–13]

On the other hand, Henriquez-Sanchez et al [14] observed a positive relationship between adherence to the MedDiet and four physical health categories of HRQL in highly educated and younger subjects in their longitudinal assessment of the SUN cohort, although there were no significant associations with most of the mental health dimensions. While the SUN study and the present one have been conducted in Spain and consider a full dietary pattern, participants in the SUN cohort were younger and healthier than those in the PREDIMED-Plus trial, who were aged between 55 and 75 years, had overweight or obesity, and all met criteria for metabolic syndrome. An average worse score in physical dimensions could also be attributable to a relatively higher prevalence of obesity, type 2 diabetes and tobacco consumption [24]. Furthermore, Perez-Tasigchana et al [7] found slightly better results in physical categories when a high adherence to Mediterranean dietary pattern [25] was reported in the ENRICA cohort, but they had a smaller sample, used a shorter version of the questionnaire and the differences were small and probably not clinically relevant.

Although current evidence suggests a positive association between adherence to the MedDiet and self-reported HRQL, the mechanisms involved are still unclear. Life domains such as social interactions, personal satisfaction or economical characteristics could have a reciprocal relationship with food habits and the way we eat and, together with the physical and psychological health, could define what we consider to be a good or poor quality of life. On the other hand, there is evidence suggesting that conformity to the MedDiet reduces the risk of chronic diseases [26] and is protective against cardiovascular diseases [2,3,27–31]. The MedDiet has also been linked to a lower risk of depression [15,32] and better cognitive function [33–35]. There is also an inverse relationship between fruit and vegetable consumption and perceived stress and depressive symptoms [36]. Therefore, it seems coherent that in overweight/obese subjects, as those in the PREDIMED-Plus trial, a better adherence to the MedDiet might be associated with better scores in the mental dimensions of HRQL.

Despite the large sample size, adjustment for a wide array of potential confounders, and inclusion of participants from various regions of Spain with different dietary habits, our study has limitations. First, the cross-sectional design does not allow to establish an appropriate temporal sequence and cannot demonstrate causality, because reverse causation cannot be ruled out. The association that we report here might be bi-directional. Second, our results stemmed from participants with overweight or obesity who had been just recruited for a trial aiming to assess the effects of an MedDiet together with promotion of physical activity and a behavioral intervention compared to a nutritional intervention aiming to increase adherence to an ad libitum traditional Mediterranean dietary pattern. Thus, the personal satisfaction of participants immediately after being enrolled into the trial might have also affected the results. Nevertheless, information on both diet and HRQL was collected at baseline, in the initial visit, before participants had been exposed to the intervention and we did not compare the intervention versus the control group in this assessment. Third, although we adjusted for many potential confounders, residual confounding might have affected the results. Fourth, information on diet was self-reported. Nevertheless, it was collected in a face-to-face interview with trained dietitians, which increases the accuracy of the information. In any case, the resulting misclassification would have biased our results towards the null. Finally, we had no information on socioeconomic status which is also related to health and self-reported health [22]. However, we adjusted for educational level, which is a proxy of socioeconomic status and is also in itself a determinant of adherence to a healthy diet and of HRQL [37].

## Conclusions

Our results suggest a positive association between higher adherence to a MedDiet and better quality of life and each of its dimensions. Since there are some differences in the results compared to previous reports, we believe that further studies, preferentially with a longitudinal design, are needed in order to better ascertain the direction of causality and to identify which dimensions of HRQL are more susceptible of improvement by following a MedDiet.

## Supporting information

S1 File Dataset. This is the dataset.
(XLSX)

S2 File Dataset. This is the dataset with which the analyses were conducted with labels.
(XLSX)
